# Characterization of the Pozzolanic Potential of Oil Palm Kernel Shell Ash Obtained Through Optimization of Physicochemical Processes

**DOI:** 10.3390/ma18061248

**Published:** 2025-03-12

**Authors:** Ramon Torres Ortega, María Luna Velasco, Jair Arrieta Baldovino

**Affiliations:** 1 Civil Engineering Program, University of Cartagena, Calle 30 # 48-152, Cartagena de Indias 130001, Colombia; jarrietab2@unicartagena.edu.co; 2 Faculty of Engineering, University of Cartagena, Calle 30 # 48-152, Cartagena de Indias 130001, Colombia; mlunav@unicartagena.edu.co

**Keywords:** oil palm kernel shell ash, X-ray fluorescence, cement, concrete, sustainability, pozzolan, chemical properties, optimal temperatures

## Abstract

Oil palm kernel shell ash (POFA), a byproduct of the highly cultivated agro-industrial sector in Colombia, has been widely used for its pozzolanic properties, which enhance the mechanical and durability characteristics of concrete. Six POFA samples were analyzed after undergoing drying, cutting, grinding, crushing, and calcination at temperatures ranging from 500 °C to 1000 °C. SEM-EDS/EDX analysis, X-ray fluorescence (XRF), and loss on ignition (LOI) tests were conducted to characterize its pozzolanic potential. The results revealed that the SiO_2_ content increased with the calcination temperature, reaching a peak of 76.8% at 1000 °C. However, calcination at 600 °C was identified as the optimal temperature, as it balances impurity removal without inducing the formation of crystalline silica, which would negatively affect the material’s reactivity. Considering the optimal calcination temperature and the high initial LOI values, which exceeded 70% in the first calcination stage, a second calcination was performed on the 500 °C sample by increasing the temperature to 600 °C. This resulted in an LOI of 3.33%, according to ASTM C311 standards for natural pozzolans used in Portland cement concretes.

## 1. Introduction

The ashes from agro-industrial byproducts, often regarded as mere waste, can actually serve as a valuable source of useful elements for various industrial and agricultural applications [1,2]. These ashes contain a variety of minerals and nutrients, such as silica, potassium, calcium, and magnesium, which can be utilized as natural fertilizers, enhancing soil quality and promoting healthy plant growth [3]. Additionally, certain components of agro-industrial byproduct ashes have applications in construction materials and ceramics, providing a sustainable alternative to traditional resources [4,5]. In this field, various types of ashes have been studied as potential cement substitutes due to their significant silica content [6].

The silica content is highly significant in pozzolans used as an additive or partial replacement for cement in concrete, as the silica present in these ashes can react with the calcium hydroxide released during cement hydration, forming calcium silicate hydrates (C-S-H) [7], which are responsible for the mechanical properties of cement [1,8]. The incorporation of agro-industrial byproduct ashes in cement and concrete manufacturing is highly beneficial for zero-waste technology and sustainable development [9]. Agro-industrial byproducts, which contain high concentrations of silica, can serve as sources of renewable energy and exhibit notable pozzolanic properties when burned under specific conditions [10]. In addition, finely ground ashes can act as filler materials, improving the packing density of concrete and, consequently, its mechanical properties and durability [11].

Palm oil production generates significant byproducts, such as palm kernel shells, the management of which represents a considerable environmental challenge [12]. Palm kernel shell ash is a byproduct that has gained attention for its pozzolanic properties, making it suitable for use as a partial replacement for cement [13]. These silica-rich ashes, when finely divided and in the presence of moisture, chemically react with the calcium hydroxide released during the hydration of Portland cement, forming calcium silicate hydrate and other cementitious compounds [14]. Previous studies have shown an improvement in compressive strength for concretes with the addition of POFA [15]. This process not only improves the strength and durability of concrete but also contributes to agricultural waste management [16]. Currently, the use of palm kernel shell ash as a partial substitute for cement, or as an additive in concrete production, is being investigated with the aim of improving its mechanical properties and promoting environmental sustainability [17].

The impact of high temperatures on the microstructure and compressive strength of self-compacting concrete with a 15% replacement level of POFA by weight of cement has been analyzed [18]. The compressive strength of the samples was evaluated at 28 days, and they were subsequently exposed to high temperatures ranging from 200 °C to 1000 °C, in 200 °C intervals. The results showed a continuous mass loss in the samples as the temperature increased and an increase in compressive strength values at 400 °C, while these fluctuated notably in the 400–600 °C, 600–800 °C, and 800–1000 °C ranges. Additionally, microstructural analyses revealed the transformation of calcium silicate hydrate (C-S-H) into distinct phases. The authors suggest that the results of this research may be applicable in structures with high fire resistance and will also contribute to minimizing the waste generated in palm oil mills [19]. On the other hand, they [20] reviewed the potential use of POFA as an alternative cementitious material in concrete, through an analysis of its impact on the properties of concrete in the fresh and hardened states, as well as its durability. They determined that the particle-grinding treatment of POFA significantly improves its quality in terms of compressive strength, resistance to aggressive environments, and reduction in drying shrinkage in concrete. However, it tends to increase water absorption and delay the heat of hydration in cement mortar [21]. Additionally, these authors determined that the high SiO_2_ content in POFA facilitates the pozzolanic reaction and delays the setting times with the addition of CaO, which leads to the formation of more C–S–H gels. They concluded that additions of 20% POFA or 30% ultra-fine and Nano POFA can produce high-strength and durable concretes, making a promising contribution to the sustainability of the construction industry.

The characterization of ash derived from palm kernel shells using X-ray fluorescence allows for the determination of the elemental composition of the ash, identifying the presence of key components, such as silica, calcium, and other metallic oxides that influence the pozzolanic and cementitious properties of the material [22]. Furthermore, X-ray fluorescence can help identify potential contaminants or trace elements that could negatively affect the performance of concrete, allowing for the development of treatment or purification methods for the ashes [23]. Ultimately, this advanced characterization not only facilitates a more efficient and effective use of agricultural waste but also contributes to the development of more sustainable and high-performance construction materials. The application of this technique not only enhances the understanding of the physical and chemical properties of the ashes but also enables standardization and quality control in their production and use [24].

In line with these considerations, other authors have investigated the properties of nano POFA (nPOFA) using X-ray diffraction (XRD), Fourier-transform infrared spectroscopy (FT-IR), scanning electron microscopy (SEM), and transmission electron microscopy (TEM), detecting that nPOFA is primarily composed of SiO_2_, with an average particle size of 100–150 nm [25]. Furthermore, the authors observed through these characterization techniques that, with increased curing time, the microstructure of specimens with nPOFA became denser compared to the samples without nPOFA, due to the refinement of the microstructure. Thermogravimetric analysis (TGA), XRD, and FT-IR confirmed the reduction in Ca(OH)_2_ due to the pozzolanic reaction and the additional formation of C–S–H gels. Therefore, the authors support the use of nPOFA as an eco-friendly cementitious material [25]. On the other hand, they [26] analyzed the effects of incorporating POFA and fly ash (FA) as partial cement substitutes on the physicomechanical properties of high-quality mortars. To do this, the authors subjected mortar samples to slump flow tests, water absorption, compressive strength testing, and characterization through X-ray fluorescence (XRF) analysis, Fourier-transform infrared spectroscopy (FTIR), scanning electron microscopy/energy-dispersive X-ray (SEM/EDX), and X-ray diffraction (XRD) [26]. Thus, the FTIR analysis confirmed the presence of SiO and AlO groups within the POFA-FA compound. The XRF analysis of FA and POFA showed cementitious properties, with a combined SiO_2_ + Al_2_O_3_ + Fe_2_O_3_ content exceeding 50% and CaO above 10%. The SEM and XRD results indicated minimal formation of cavities, suggesting high compressive strength in the concrete. The particle size distribution analysis revealed that most particles were in the range of 0.15 to 0.2 μm. The compressive strength test after 28 days, with the incorporation of 15% FA and 10% POFA, showed the highest strength, reaching 59.30 MPa. This is attributed to the high density of the material, which leads to the formation of fewer voids, reducing trapped water that could significantly affect the mortar’s strength.

In this context, it should be mentioned that the partial replacement of cement with supplementary cementitious materials (SCMs) has become a solution to mitigate the issues associated with concrete production. For this reason, recent studies have explored the use of agro-industrial and construction waste as partial replacements for cement or aggregates in different types of concrete. Thus, it is relevant to discuss geopolymers, which are alkali-activated binders with low calcium content [27] and slags, which have high calcium content and are used in alkali-activated materials (AAMs). Slags consist of a vitreous substance called “melilite”, which depolymerizes under the action of alkalis in an alkaline environment. A challenge with AAMs is that the alkali cations (K^+^ and Na^+^) remain outside the reaction cycle and can leach out and migrate when in contact with water. Therefore, to stabilize the structure of alkali-activated slag (AAS), a structuring element such as metakaolin or fly ash must be added, as they react with the free cations mentioned.

On the other hand, geopolymers are produced by dissolving an aluminosilicate oxide into an alkaline solution, typically consisting of sodium hydroxide, a combination of sodium hydroxide and sodium silicate, or potassium hydroxide with potassium silicate. The polymerization between Al and Si complexes and the alkaline solution leads to the formation of three-dimensional polymeric Si-O-Al chains [28]. Thus, AAMs can incorporate various industrial residues as precursors, whereas geopolymers primarily rely on specific materials rich in Si and Al compounds, such as oil palm kernel shell ash [29].

This study aims to characterize palm kernel shell ash and evaluate its elemental composition, with a particular focus on silica (SiO_2_) and carbon content at various temperature processes. Three key techniques were employed: X-ray fluorescence (XRF), scanning electron microscopy (SEM), and loss on ignition (LOI) determination. The XRF was essential for determining the elemental composition of the samples, specifically to identify the silica content and other reactive compounds critical to the pozzolanic properties. Meanwhile, SEM allows for the analysis of the morphology and texture of the particles, which is crucial for understanding how the material’s structure affects its reactivity and behavior for use in cement mixtures. Additionally, the SEM analysis enabled the evaluation of the carbon content present in the samples studied and the correlation of this content with the LOI results. Based on these material characterization techniques, the optimal temperature processes for obtaining palm kernel shell ash with pozzolanic potential for use in concrete will be determined, validating the physicochemical processes obtained [30]. The main contribution of this article is to characterize the potential of oil palm kernel shell ash for use as a pozzolan in concrete. To achieve this, starting from the physicochemical processes experimentally demonstrated as optimal in [30], an XRF analysis is conducted at different calcination temperatures, selecting the one that achieves the highest amorphous silica content, which is essential for reacting with cement and forming C-S-H gel. However, the LOI analysis will determine whether these thermal processes are sufficient for pozzolanic classification based on the organic material content present in the ash. Subsequently, final SEM and XRF tests will be conducted to characterize the definitive material obtained. Ultimately, this article aims to establish a standardized process for obtaining oil palm kernel shell ash that meets ASTM C618 standards for use as a cement replacement in concrete.

## 2. Materials and Methods

### 2.1. Sample Preparation

**Figure 1:**
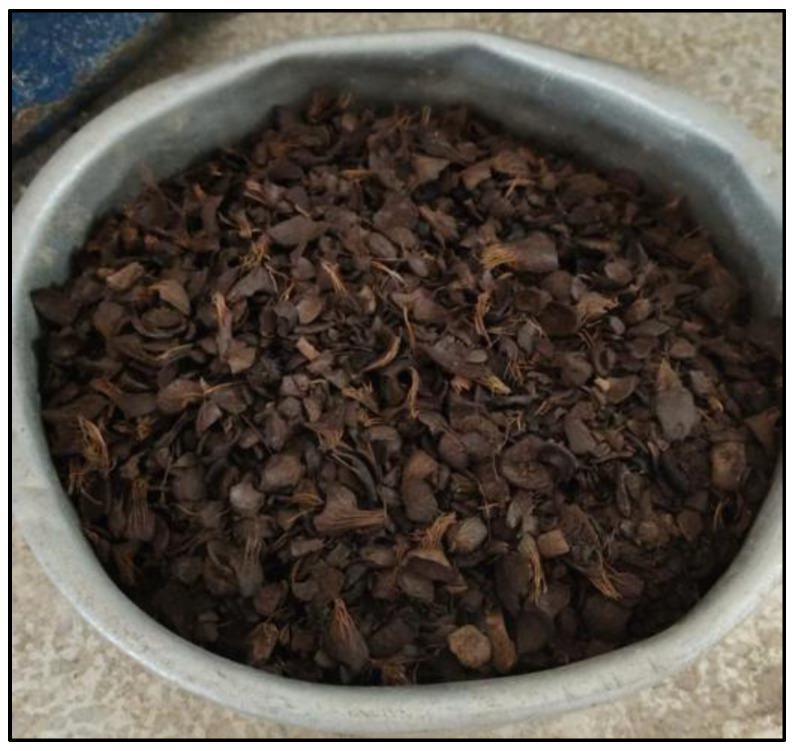
Dehydrated oil palm kernel for 24 h at 100 °C. (Reprinted from Ref. [30]).

Palm kernel shell (POFA) samples were collected from a palm-kernel-processing plant in the northern region of the Bolívar department, Colombia. In the laboratory, a dehydration process was carried out at 100 °C for 24 h in an oven for all the palm kernel shell samples, as shown in Figure 1.

**Figure 2:**
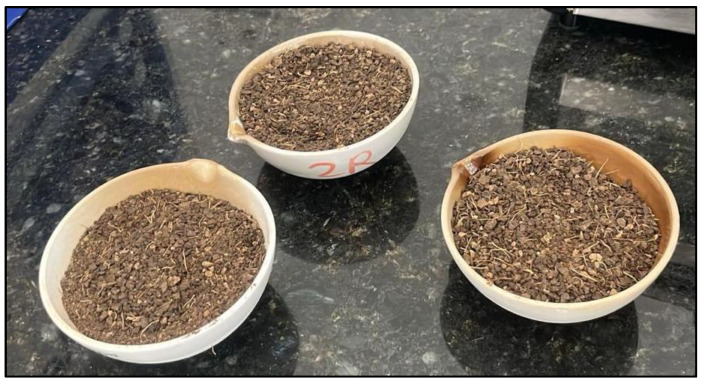
Dehydration at 100 °C, cutting of the kernel, and grinding in a manual mill.

Subsequently, after the dehydration process, the palm kernel shells were fragmented using an electric machine that performs cutting solely due to the nature of the material. In the second process, after cutting, the samples were subjected to grinding using a manual mill. This process is shown in Figure 2.

**Figure 3:**
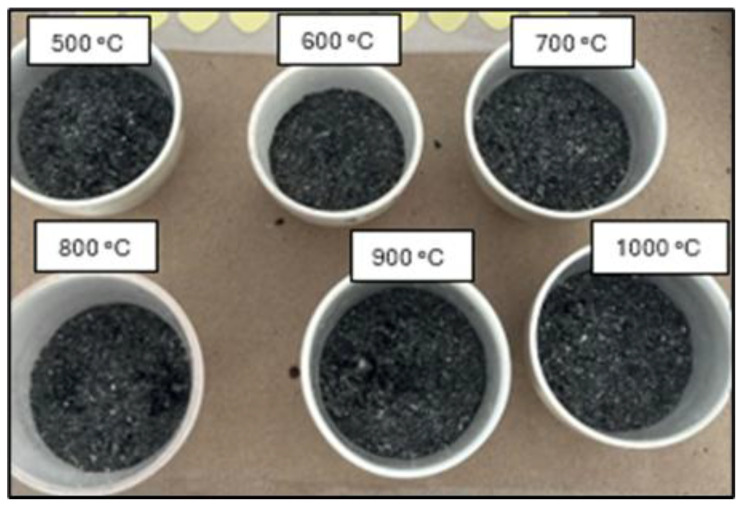
Palm oil cores with physical processes of cutting using an electric machine and grinding in a manual mill and calcining in a muffle at different temperatures. (Reprinted from Ref. [30]).

**Figure 4:**
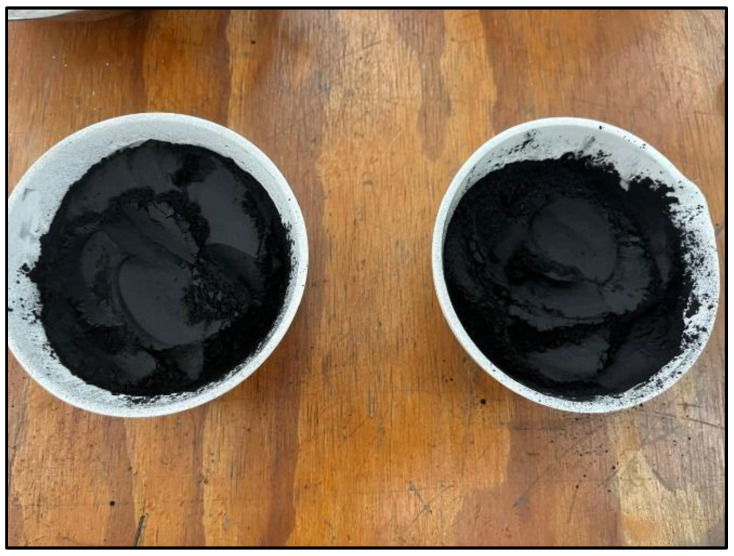
Sample with dehydration at 100 °C, kernel cutting, grinding in a jaw crusher, calcination at 500 °C, and grinding in a ball mill. (Reprinted from Ref. [30]).

The samples were then subjected to a calcination process at varying temperatures between 500 °C and 1000 °C for 1.5 h in a muffle furnace, and finally, they underwent crushing in a ball mill for 1 h, as shown in Figure 3 and Figure 4.

In Table 1, the respective processes performed on each sample and their corresponding calcination temperatures are shown, assigning a specific name to each, expressed in an abbreviated form to represent each of these processes.

### 2.2. Testing

**Figure 5:**
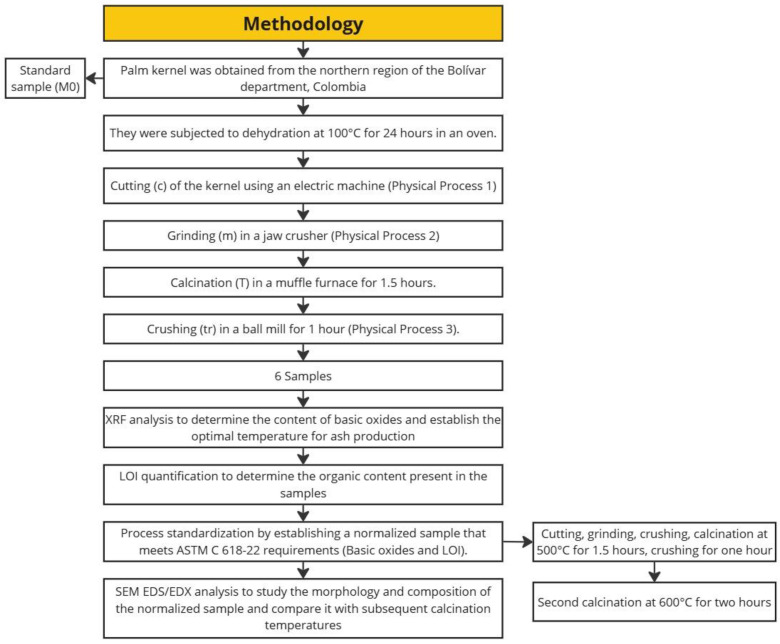
Experimental methodology.

For the characterization of the pozzolanic potential of palm kernel shell ash, a total of six samples were analyzed using X-ray fluorescence (XRF), loss on ignition (LOI) determination, and scanning electron microscopy (SEM), complemented with energy-dispersive X-ray spectroscopy (EDS), as shown in Figure 5.

X-ray fluorescence (XRF) is a powerful analytical technique that enables precise and non-destructive characterization of materials [31]. In the case of ashes derived from palm kernel shells, the use of XRF can provide a detailed quantification of the elements present, from the major components to trace elements [32]. This information is crucial for understanding how each component contributes to the overall properties of the ashes and, consequently, to the concrete in which they are incorporated [33]. By integrating XRF techniques into the research and development of construction materials, potential contaminants can be efficiently identified and mitigated, and waste reutilization can be optimized, thus promoting more sustainable practices in the industry [34]. The XRF technique is suitable for determining the thickness and composition of films, as well as for qualitative and quantitative elemental analysis [35].

The chemical composition of POFA samples calcined at different temperatures was determined using a sequential wavelength dispersive X-ray fluorescence (XRF) spectrometer. The results were expressed as weight percentages of the oxides present in the samples. The XRF test will be used to determine the optimal calcination temperature for obtaining palm oil kernel ash. Additionally, by calculating the loss on ignition (LOI), the organic content of each sample will be determined to assess its potential as a pozzolanic material in concrete. This is based on the consideration that high LOI percentages indicate active organic content in the ash, which can negatively affect the mechanical strength and durability of concrete. It is known that LOI values higher than 10% significantly reduce early-age compressive strength, while LOI values below 10% provide excellent pozzolanic material with potential for partial replacement of Portland cement in concrete [36]. Finally, the SEM analysis provides a powerful tool for the detailed characterization of samples, as it offers high-resolution images that allow for an in-depth examination of particle morphology and the determination of elemental composition. The combined use of SEM-EDS and SEM-EDX ensures a comprehensive evaluation of the physical and chemical properties of the ashes, facilitating a deeper understanding of their potential as a cementitious material [30]. It is important to note that a thermionic emission source device was used for the SEM analysis, with an acceleration voltage ranging from 0.3 to 30 kV. Additionally, the EDS microanalysis system used was the INCA PentaFETx3 from Oxford Instruments.

## 3. Results and Discussion

### 3.1. X-Ray Fluorescence (XRF)

In Table 2, the results of the chemical composition, expressed as weight percentages, of the analyzed samples are presented. Previous studies have highlighted that the pozzolanic reaction is activated when the ash contains an adequate concentration of silicon dioxide (SiO_2_), aluminum oxide (Al_2_O_3_), and iron oxide (Fe_2_O_3_) [37,38]. As shown in Table 2, this increase in the concentration of SiO_2_ as the calcination temperature rises implies a higher potential for the ashes to be used as a partial substitute for cement in the production of high-strength and durable concrete, positioning POFA as a viable option in terms of sustainability and environmental impact reduction.

The chemical composition results of each evaluated sample were compared with the requirements of the ASTM C 618 standard for pozzolans. The analyzed samples meet the chemical composition requirements for Class N pozzolans according to the standard [39]. This indicates that they are either raw or calcined natural pozzolans and materials that require calcination to induce satisfactory properties. According to the [39], the chemical composition requirement for basic oxides—silicon dioxide (SiO_2_), aluminum oxide (Al_2_O_3_), and iron oxide (Fe_2_O_3_)—is a minimum of 70% for Class N pozzolans to be used in concrete. Upon analyzing the results obtained, it can be observed that, for all the tested samples, including sample 0 (MO-T100C-C-t24 h-c-m), this requirement is met. Additionally, the maximum allowed percentage of sulfur trioxide (SO_3_) for Class N pozzolans is 4%, according to the standard [39]. And all the analyzed samples are within this limit, confirming their compliance with the quality standards. It should be noted that the limits on the content of these chemical compounds arise because the specifications allow the use of ashes produced from coal combustion with co-combustion materials, as is the case with oil palm kernel shell ash. Consequently, elements such as free P_2_O_5_ and CaO in this type of supplementary cementitious materials are known to cause significant setting delay and expansion, respectively [40]. However, the content of these elements in all the studied samples is below 5%, suggesting that their presence will not be detrimental when incorporating these ashes into cementitious matrices.

**Figure 6:**
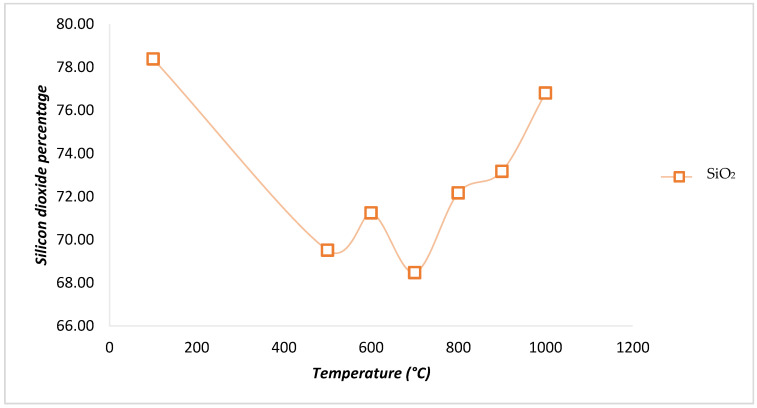
Graph of silicon dioxide (SiO_2_) percentage versus temperature for the samples.

Figure 6 illustrates the variation in the percentage of silicon dioxide (SiO_2_) in the evaluated samples as a function of the calcination temperature. It is observed that the uncalcinated sample (M0, dried at 100 °C) exhibits the highest silica content, exceeding 78%, indicating that the oil cake has a high potential as a pozzolanic material, given that the presence of silica in pozzolanas is crucial for its reaction with Portland cement and the subsequent formation of CSH gel. For the sample M17A-c-m-T500C-t1.5h-tr1h subjected to 500 °C, a decrease in the silica content is observed, reaching 69.51%, suggesting the combustion of silica-rich particles. However, the sample calcined at 600 °C (M18A-c-m-T600C-t1.5h-tr1h) shows an increase in silica content, peaking at 71.24%. Therefore, this temperature is considered optimal, since, at 700 °C, a decrease in the silica content occurs, and temperatures above 800 °C cause a phase change from amorphous to crystalline, which is undesirable for pozzolanas added to concrete [30,41]. Thus, it is observed that combustion at 700 °C promotes the burning of silica-rich particles, which reduces the SiO2 content at this temperature compared to 600 °C and 800 °C.

Previous studies have shown that, in untreated samples, the silica content may appear higher due to the presence of organic and carbonaceous material that has not been removed [38,42]; this can retain fine silica particles in their natural state. However, this silica content may not be fully available for the pozzolanic reaction due to the interference of these organic compounds [43]. Therefore, although the SiO_2_ percentage may appear high, its reactivity is limited until the material undergoes a calcination process [44].

Based on the results indicated in Table 2 and the graph obtained from the comparison of these results with respect to the temperature (Figure 6), it becomes evident that, for the calcined samples, as the temperature increased, the silica content also increased. However, the temperature of 600 °C is considered optimal, as it is higher than both 500 °C and 700 °C, and it is noted that, according to the literature, temperatures above 800 °C promote the formation of crystals, causing silica to transition from amorphous to crystalline [40], which can alter the material’s composition [45]. This transformation is critical, as crystalline silica is less reactive as a pozzolan, reducing its effectiveness in cementitious applications [46]. Additionally, it has been experimentally demonstrated that a temperature of 600 °C allows for a higher silica content and exhibits lower carbon contents [30]. This temperature was selected due to its potential for use as pozzolans and because it yields a greater amount of pozzolana than temperatures higher than 600 °C.

In general, palm oil kernel ash (POFA) has a silica (SiO_2_) content comparable to or even higher than other agricultural ashes analyzed, especially at higher calcination temperatures. Although POFA presents a favorable chemical profile, other types of ashes may offer similar or superior properties depending on their specific composition and the thermal treatment applied. Furthermore, the improvement in the compressive strength of concrete incorporating these ashes, compared to control mixes, highlights their potential as a partial cement substitute, offering a sustainable alternative in construction [47].

The result of the chemical analysis of palm oil kernel ash (POFA) was compared with various studies that have investigated the potential use of ashes from different agricultural products in concrete, such as rice husk ash (RHA) [48], olive seed ash (OSA) [49], ceniza de biomasa de astillas de madera (BA) [50], biomass ash from wood chips (BA) [9,51], and bagasse ash from sugarcane (SCBA) [52,53]. The results of this comparison indicated POFA as a good pozzolanic material due to its high silica content, as well as WSA and SBA. The results of the RHA showed a silica content higher than that of POFA [48,54].

Below is a comparison between POFA-FB, which refers to the fruit bunch ash of the oil palm, POFA-K, understood as the ash from the oil palm kernel shell, and G-POFA, corresponding to the ground ash of the oil palm kernel. Additionally, a comparison is made with studies addressing U-POFA, which refers to the ultrafine ash of the oil palm kernel, T-POFA, characterized as the treated ash of the oil palm kernel, MT-POFA, referring to the treated and modified oil palm kernel ash, and POCP, corresponding to the powder of the oil palm kernel.

The results of the study [55]: The results of the study for POFA-FB and POFA-K show a SiO_2_ content of 64.72% and 64.07%, respectively, at calcination temperatures of 60 °C and 150 °C, as detailed in Table 3. These results do not meet the minimum requirement of 70% in the sum of SiO_2_, Al_2_O_3_, and Fe_2_O_3_ contents to be considered effective pozzolans according to ASTM C618. In contrast, the palm kernel ash in this study meets the regulatory requirement when calcined at higher temperatures, indicating that a higher calcination temperature improves the silica availability and, therefore, the pozzolanic quality of the ashes, as long as the maximum temperature limit is not exceeded.

The palm kernel ash (POFA) samples at temperatures between 500 °C and 1000 °C show a SiO_2_ content ranging from 68.47% to 76.80%, thus meeting the minimum requirement of 70% for the sum of SiO_2_, Al_2_O_3_, and Fe_2_O_3_. In terms of comparison with previous studies, a clear difference in SiO_2_ content values is observed. The results reported in the literature for samples calcined at 600 °C reflect a significant difference in the methodology applied and its implications on the chemical composition of the ashes. Thus, the existing literature reports significantly lower silica contents, with most values below 65.01%, whereas, in this study, the samples characterized experimentally using XRF exhibit much higher silica content, showing a notable disparity with these results. For the ultrafine sample (U-POFA) and treated modified samples (MT-POFA) calcined at 600 °C, these silica values were higher, approaching the values from the present study. The contrast can be explained by the additional processes applied in this research, such as cutting, crushing, and grinding, which improve the fineness of the material and, therefore, increase the reactive surface area, optimizing the release of silica.

In this research, the additional processes of cutting, crushing, and grinding played a key role in improving the pozzolanic properties. Crushing and grinding help reduce the particle size of POFA, increasing the specific surface area available to react during cement hydration. Additionally, it promotes a greater release of silica, as the internal layers of the material are exposed to the calcination and crushing processes. This study demonstrates that the application of cutting, crushing, and grinding processes, combined with calcination at 600 °C, maximizes the availability of silica, improving POFA’s properties as a pozzolanic material and suggesting that these methodologies should be followed to achieve optimal results in cementitious applications.

In Table 3, it is observed that the U-POFA samples U-POFA [56,57], the T-POFA sample [37,58], and the MT-POFA sample [61] meet the minimum requirement of 70% according to ASTM C618 for the sum of the oxides SiO_2_, Al_2_O_3_, and Fe_2_O_3_, with the MT-POFA sample, calcined at a higher temperature, exhibiting the highest silica content. This indicates that both the calcination temperature and the material fineness are critical factors that must be carefully controlled to achieve optimal results. It can also be observed that the two samples that do not have calcination data do not meet the criteria to be considered pozzolanic materials. Therefore, it would be beneficial to investigate the impact of different grinding methods, as, as observed, the fineness of the material improves its pozzolanic efficiency by increasing the reactive surface area. Hence, a combination of calcination at optimal temperatures (around 600 °C) and advanced grinding techniques can maximize the performance of POFA ashes as a partial cement replacement material.

It can also be observed, upon making the comparison, that the samples that do not meet the minimum requirement of 70% for the sum of SiO_2_, Al_2_O_3_, and Fe_2_O_3_ have only one grinding or crushing process, whereas those that do meet the requirement mostly have more than one process. For example, U-POFA and T-POFA have an additional grinding process, and the samples from this study undergo three processes. As a future recommendation, it is urged to conduct a detailed evaluation of the properties of the soil in which the oil palm is grown to determine its impact on the silica content of the kernel ashes. Soil quality can significantly influence the silica content and other compounds in the ashes, which would, in turn, affect their pozzolanic reactivity [68], since the soil pH affects the solubility of silicon in it, facilitating the absorption of silica by the crops [69]. Similarly, the texture and the presence of organic matter in the soil can affect the availability of silica contained in it, and salinity can impact the absorption of nutrients by the crops [68].

### 3.2. Loss on Ignition (LOI)

The calcination of biomass ashes such as POFA becomes necessary primarily to reduce the loss on ignition (LOI), which corresponds to the content of unburned carbon and other organic impurities that do not contribute to pozzolanic activity. The current standards, represented by ASTM C-618 [39], set a maximum LOI limit of 10% for Class N pozzolans to be suitable for use in concrete, as higher values negatively affect the reactivity of the material and, consequently, the mechanical properties of the concrete. A high LOI value is indicative of unburned carbon, which interferes with the hydration reaction and increases the water demand, affecting the final properties of the concrete. In the case of POFA, the calcination process helps reduce these undesirable components, improving the purity of the material and activating SiO_2_ for the formation of calcium silicate hydrates (C-S-H). 

The procedure of ASTM C311 standard [45]: It sets a temperature of 750 °C ± 50 °C for natural pozzolans as the standard to ensure consistent results in the LOI measurement without significantly degrading other important components of the material. If the temperature is too high, it may affect the composition of the remaining materials, altering the number of oxides and other critical components in the analysis.

Based on the literature reviewed and referenced in Table 3, the samples that do not present calcination data [38,42] showed a loss on ignition of 20.7% and 15%, respectively, well above the limit specified by ASTM, suggesting a significant amount of carbonaceous and non-reactive materials. As the calcination temperature increases, a significant reduction in LOI is observed, with values ranging from 1.8% at 500 °C to 6.19% from 800 °C to 1000 °C for the POFA samples with various grinding processes [37,56,64]. These results confirm that calcination is necessary not only to remove unburned carbon but also to improve the reactivity of SiO_2_ and ensure the effectiveness of the material as a pozzolan in concrete production.

Table 4 shows the relationship between the calcination temperatures of the POFA samples calcined at temperatures ranging from 500 °C to 1000 °C and the losses associated with the organic material, primarily represented by the carbon present in the samples.

**Figure 7:**
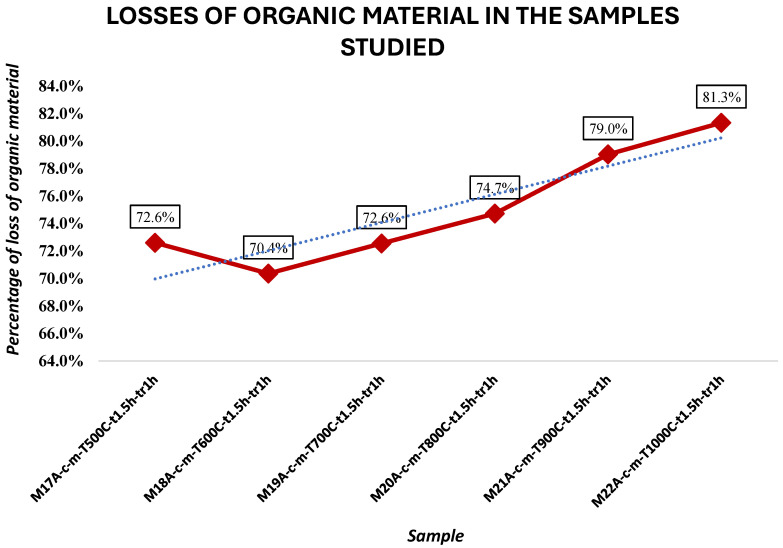
Losses in organic material content mainly associated with carbon loss in each of the studied samples.

Below is a graphical representation of the variation in organic material content losses for the different samples analyzed, as show in Figure 7. It can be observed that sample M0, which undergoes drying at 100 °C instead of calcination, exhibits a moisture content of 14.5%. In contrast, sample M17A, subjected to calcination at 500 °C, experiences organic material losses reaching 72.60%. Notably, there is a decrease in organic material losses for the sample calcined at 600 °C (M18A), dropping to 70.36%, making it the sample with the lowest recorded losses. From this point onward, a linear relationship between calcination temperature and organic material loss percentage becomes evident, indicating that higher temperatures result in greater losses.

As can be seen, the percentage of weight loss due to organic material content is very high. This can be verified by the color of the samples. (Figure 4); the samples are black in color, indicating a high carbon content. Additionally, it should be noted that the sample **M18A-c-m-T600C-t1.5h-tr1h** has the lowest percentage of organic material losses, establishing that this temperature allows for the burning of a higher carbon content compared to the other samples evaluated. However, the percentage of organic material losses remains very high. Therefore, it was decided to subject the sample to calcination once again.

**Figure 8:**
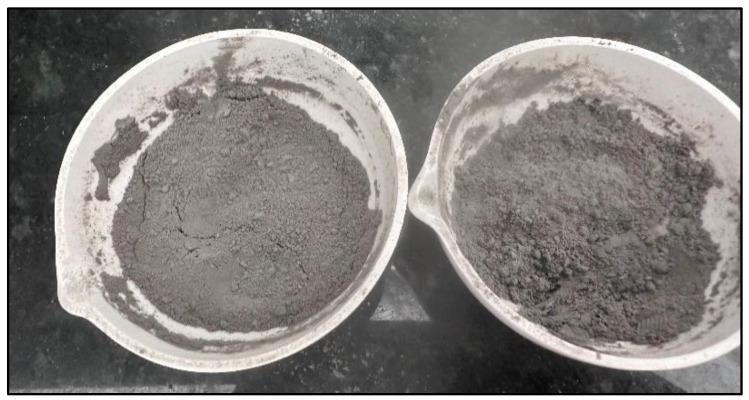
Final sample after the processes of cutting, grinding, calcination at 500 °C, crushing, and a second calcination at 600 °C.

**Figure 9:**
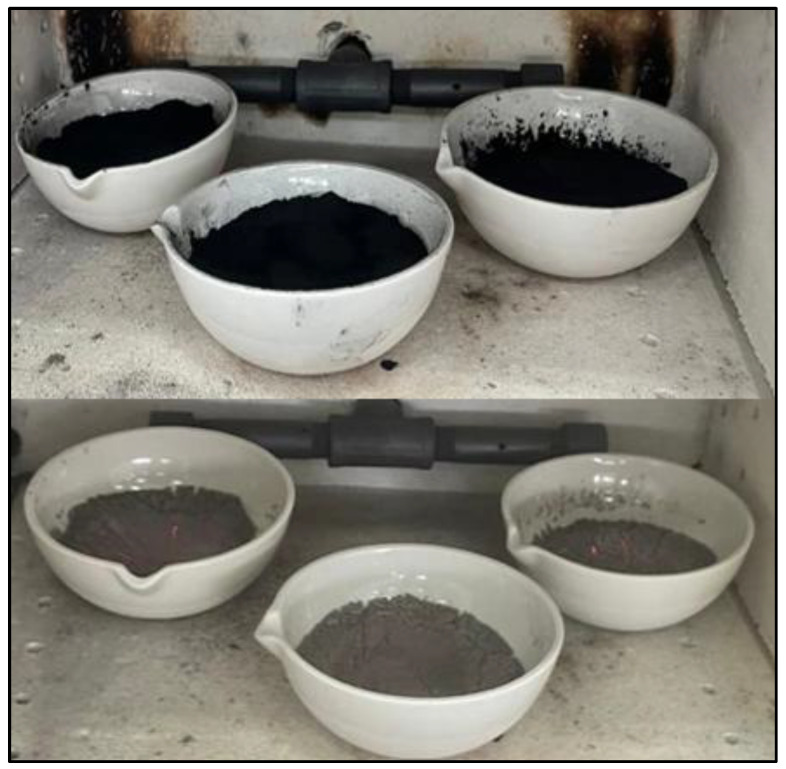
Comparison between M18A-c-m-T600C-t1.5h-tr1h and the final sample MDEF-c-m-T500C-t1.5h-tr1h-T600C-t2h.

For this process, the optimal temperature of 600 °C, previously established based on the results obtained through XRF, was considered. Therefore, the sample initially calcined at 500 °C (considered the temperature at which the combustion of the oil palm seed begins) was subjected to a second calcination at 600 °C for 2 h, with stirring to incorporate the material, followed by an additional 30 min of calcination, resulting in the final sample **MDEF-c-m-T500C-t1.5h-tr1h-T600C-t2h**. It should be mentioned that the samples were saved after the second combustion to obtain a homogeneous particle size. After this process, a color difference can be observed (Figure 8), compared to the samples before this process (Figure 9).

Below is a comparative image between the samples M18A-c-m-T600C-t1.5h-tr1h and the final sample MDEF-c-m-T500C-t1.5h-tr1h-T600C-t2h, where it can be observed that with the second calcination at 600 °C, the characteristic black color associated with high carbon content is eliminated.

The LOI was calculated according to the ASTM C311 standard for the final sample, which underwent cutting, grinding in a jaw mill, burning at 500 °C, crushing in a ball mill, and subsequent calcination at 600 °C (**MDEF-c-m-T500C-t1.5h-tr1h-T600C-t2h**). To eliminate residual organic material, for the LOI determination, the final sample was heated to 750 °C, as it is natural pozzolan (Equation (1), loss on ignition (LOI)).

(1) $$LOI=\frac{A}{B}\times 100$$

where

A = Mass loss between 105 °C and 750 °C

B = Mass of the sample without moisture used.

In this way, 15 g of the final POFA samples were taken (**MDEF-c-m-T500C-t1.5h-tr1h-T600C-t2h**) with calcination at a temperature of 500 °C followed by 600 °C, and the sample was subjected to a muffled furnace at 750 °C for 1 h. The final weight of the sample was 14.5 g, resulting in a loss on ignition (LOI) of 3.33%, significantly decreasing. This LOI value is within the limits allowed by ASTM C618, indicating that the oil palm seed ashes obtained through the three applied physical processes (cutting, grinding, and crushing) and the two thermal processes (calcination at 500 °C and subsequent calcination at 600 °C) can potentially be used as pozzolanic material in concrete. The obtained results highlight the importance of calcination, particularly at this temperature, as thermal processes allow for the removal of organic material. The significance of removing organic material is that it does not react properly with cement during the hydration process. Therefore, the decomposition of organic materials within the concrete can release gases or leave voids, leading to the formation of cavities that weaken the structure and may increase permeability [70]. This prevents the proper formation of hydration products, such as calcium silicate hydrates (C-S-H), which are responsible for the mechanical strength of concrete.

It should be mentioned that, in addition to the calculation of the loss on ignition at 750 °C as stated by the ASTM C311 standard, losses at 650 °C were also determined, resulting in a loss on ignition percentage of 4.4%. This confirms that the two calcination processes contribute to the removal of most of the organic material present in the sample.

### 3.3. Scanning Electron Microscopy (SEM) (SEM EDS/EDX)

**Figure 10:**
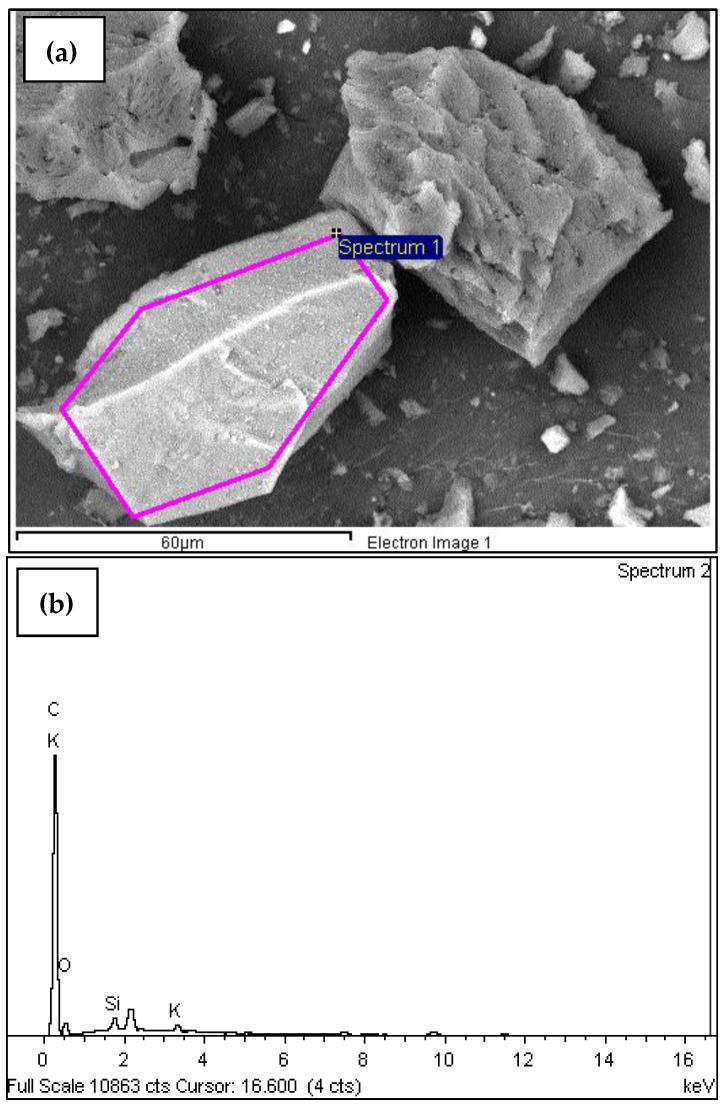
(**a**) Micrograph of the sample M18A-c-m-T600C-t1.5h-tr1h, (Reprinted from Ref. [30]); (**b**) energy (keV) of the X-rays emitted by the chemical elements that make up the M18A-c-m-T600C-t1.5h-tr1h.

A scanning electron microscopy (SEM) and energy-dispersive X-ray (EDX) analysis was performed on the sample M18A-c-m-T600C-t1.5h-tr1h. This technique allows for high-resolution images of a sample’s surface using an electron beam. It is used to analyze nanoparticles of various materials and can enhance manufacturing methods, purification systems, and make improvements in the medical industry, among others [71]. SEM allows for the detailed study of morphology and composition of materials. After performing this analysis, the sample M18A-c-m-T600C-t1.5h-tr1h showed a predominant peak of carbon (C) compared to the other elements, with a significant content of oxygen (O). (Table 5 and Figure 10b). This indicates that, with just a single calcination process at 600 °C, there is still a considerable amount of carbonaceous material and residues that have not been fully eliminated, which affects the quality of the ashes as pozzolan.

In Figure 10b, the clear difference in the intensity of the carbon peaks compared to silica (Si), potassium (K), magnesium (Mg), and calcium (Ca) is observed after performing the SEM analysis on the sample M18A-c-m-T600C-t1.5h-tr1h. This visually reveals the high organic material content present in this sample and highlights the need for a new process to eliminate it, thereby aiming to improve POFA for replacing ordinary cement. In Figure 10a, the spectrum obtained through the SEM analysis of the sample M18A-c-m-T600C-t1.5h-tr1h is presented.

**Figure 11:**
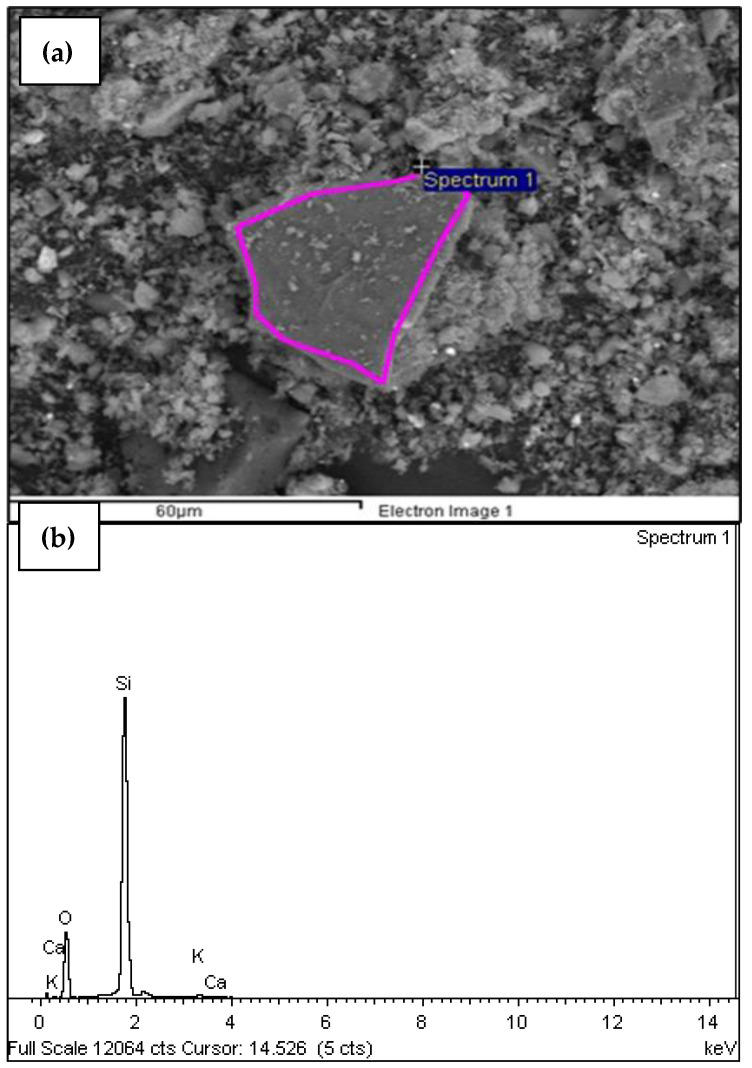
(**a**) Micrograph of the sample MDEF-c-m-T500C-t1.5h-tr1h-T600C-t2h; (**b**) energy (keV) of the X-rays emitted by the chemical elements that compose the sample MDEF-c-m-T500C-t1.5h-tr1h-T600C-t2h.

The sample M18A-c-m-T600C-t1.5h-tr1h has an average carbon content greater than 82% (Table 5); this suggests a high presence of unburned organic material. It indicates that the complete elimination of volatile compounds has not been achieved. Once the process was standardized and it was determined that the oil palm seed ash should be obtained as a potential pozzolanic material for concrete through the following processes: cutting, grinding, burning in a muffle furnace for 1.5 h at 500 °C, crushing in a ball mill, and burning in a muffle furnace for 2 h at 600 °C, SEM tests were performed again on this sample (MDEF-c-m-T500C-t1.5h-tr1h-T600C-t2h) as well as SEM tests for samples subjected to subsequent temperatures of 650 °C and 750 °C for loss on ignition determination. The SEM analyses showed the elimination of carbon in the sample and an increase in silica content Table 6 and Figure 11b.

In Figure 11a, the spectrum of the sample with the standardized process is shown. When compared to the spectrum of the sample M18A-c-m-T600C-t1.5h-tr1h (Figure 10a), there is a noticeable difference in particle size and uniformity achieved with the standardized process. It can be established that the second calcination allows us to produce smaller ash particles, facilitating their incorporation and mixing in the concrete. This results in better filling of voids, reducing concrete porosity and, consequently, enhancing its resistance to the attack of aggressive substances, such as chlorides and sulfates.

Similarly, the SEM analysis was also performed on the samples calcined at 650 °C and 750 °C after the standardized process for LOI determination. This last test was conducted to observe the behavior of the POFA based on the standardized process and its increase or decrease in carbon and silica content.

**Figure 12:**
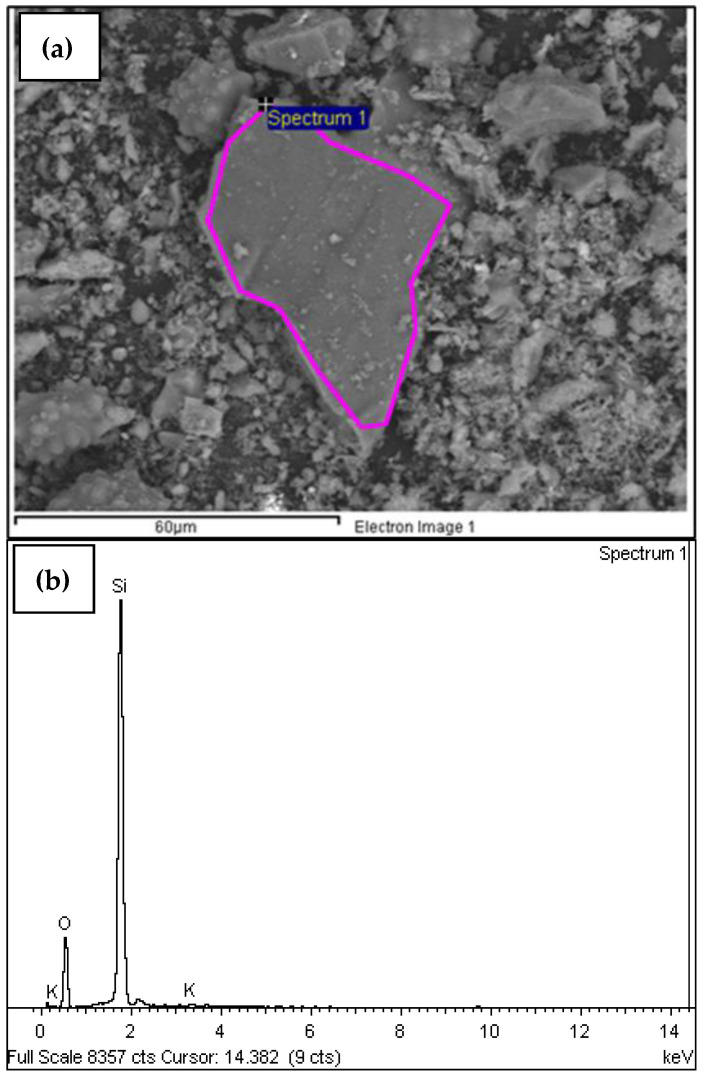
(**a**) A micrograph of the sample with the standardized process and calcination at 650 °C for LOI analysis; (**b**) a graph of the energy (keV) of the X-rays emitted by the chemical elements that compose the sample with the standardized process and calcination at 650 °C for the LOI analysis.

In Table 7 and Figure 12b, the weight percentage and the intensity graph of elements for the standardized sample are observed (**MDEF-c-m-T500C-t1.5h-tr1h-T600C-t2h**), calcined at 650 °C, to evaluate the loss on ignition at this temperature. In Figure 12a, the spectrum of this sample is shown.

**Figure 13:**
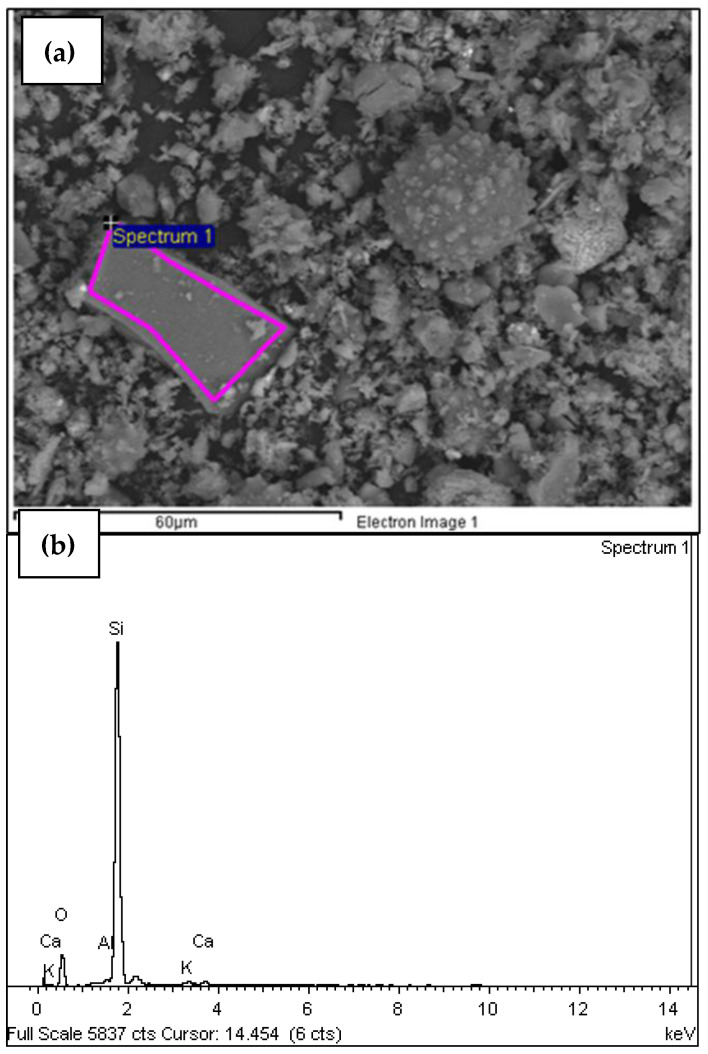
(**a**) A micrograph of the sample with the standardized process and calcination at 750 °C for LOI analysis; (**b**) a graph of energy (keV) of the X-rays emitted by the chemical elements that compose the sample with the standardized process and calcination at 750 °C for LOI analysis.

When comparing the chemical composition of the standardized sample **MDEF-c-m-T500C-t1.5h-tr1h-T600C-t2h** with that obtained for the same sample subject to an additional temperature process at 650 °C for LOI evaluation, an increase in silica content is observed. This confirms that the variation in silica concentrations indicates that the thermal conditions during the calcination process have a direct effect on the redistribution and reaction of the silica components present in the samples [30]. In Table 8, the content of the elements in the sample with the standardized process, subsequently calcined at 750 °C to evaluate LOI, is shown in weight percentage. In Figure 13a,b, the intensity of the elements and the spectrum are shown, respectively, for this sample.

**Figure 14:**
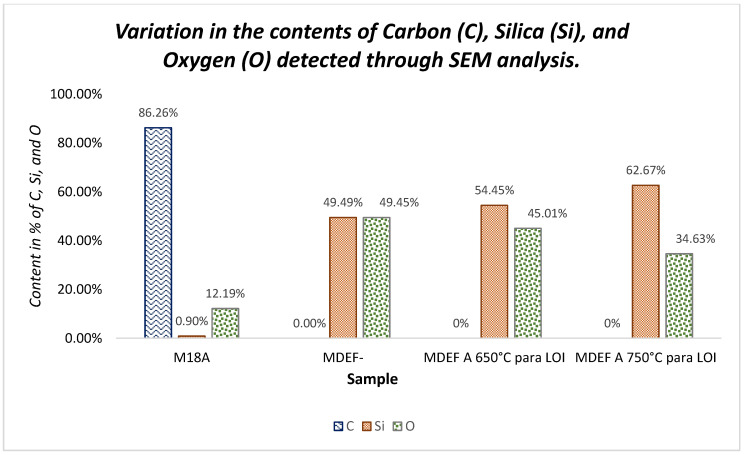
Variation in contents of carbon (C), silica (Si), and oxygen (O) detected through SEM analysis.

By comparing the elemental analysis for the sample with the standardized process and the samples with the standardized processes followed by burning at 650 °C and 750 °C, it can be observed that the silica content increased, with percentages of 49.49%, 54.45%, and 62.67%, respectively. Additionally, no carbon content is observed for these three samples. The following figure more clearly shows this variation, indicating that for the sample with a second calcination (**MDEF-c-m-T500C-t1.5h-tr1h-T600C-t2h**), transitioning from 500 °C to 600 °C, no carbon content is exhibited, in contrast to M18A (**M18A-c-m-T600C-t1.5h-tr1h**), which underwent a single calcination process at 500 °C and shows a carbon peak of 86.26%, as show in Figure 14.

These results are consistent with the LOI results, which showed that as the calcination temperature increases (up to 750 °C), the LOI percentage decreases, indicating a reduction in carbon content. It can also be stated that due to the increase in silica, the reactivity of these samples improves, which further enhances their properties as potential pozzolans. Following these considerations, it is important to mention that as the carbon content decreases with the different thermal processes, the silica (Si) content increases because of the combustion of organic material.

Finally, the XRF analysis results obtained for the sample **MDEF-c-m-T500C-t1.5h-tr1h-T600C-t2h** are presented in Table 9.

Then, from the table above, it is evident that the sample with the standardized process meets the requirements of the ASTM C618 standard, achieving a content of basic oxides of 75%, demonstrating its potential as a pozzolanic material. Thus, the importance and need to continue exploring this material to demonstrate its pozzolanicity through physical and chemical characterization techniques is clear. It should be mentioned that although the silica (SiO_2_) content in the final sample (MDEF-c-m-T500C-t1.5h-tr1h-T600C-t2h) decreases by 2.94% and 4.67% compared to the values obtained for samples M17A-c-m-T500C-t1.5h-tr1h and M18A-c-m-T600C-C-t1.5h-tr1h, the basic oxide content in the final sample still meets the ASTM C618 requirements, confirming its potential pozzolanic use. It is crucial to note that despite this slight decrease in SiO_2_ content, the reduction in organic material losses is significant and highly favorable. Therefore, it can be stated that this second calcination process at 600 °C is necessary to confer pozzolanic properties to the oil palm kernel shell ash in compliance with the current standard.

## 4. Conclusions

This study confirms that ashes derived from palm kernel shells, when subjected to a high-temperature calcination process, have a high content of silicon dioxide (SiO_2_), granting them satisfactory pozzolanic properties in accordance with the ASTM C618 standards for class N pozzolans. The analyses carried out using X-ray fluorescence (XRF) show that these ashes not only meet the minimum chemical requirements but that their silica content tends to increase with calcination temperature, suggesting higher reactivity and, therefore, greater potential to improve the mechanical properties and durability of the concrete in which they are incorporated. However, it was also found that the temperature of 600 °C provides the best results in terms of reactivity and sustainability, as this range is the most favorable for obtaining ashes with low ignition loss and high pozzolanic reactivity. Furthermore, the LOI decreases for the 500 °C sample with the new calcination process at the optimal temperature (600 °C), reaching optimal values of 3.33%, indicating effective impurity removal without compromising the pozzolanic reactivity of the material.

This finding supports the technical feasibility of these ashes as a supplementary material in concrete production and highlights their potential to enhance environmental sustainability by reducing reliance on conventional materials, minimizing agricultural waste, and decreasing CO_2_ emissions associated with cement manufacturing.

Based on the results obtained in this research, and after determining that the studied ashes have significant pozzolanic potential, further investigation will be conducted to expand this line of research. This will involve performing tests to assess pozzolanic activity using techniques such as the mechanical method and the Frattini method. Additionally, it is essential to carry out a comprehensive economic and environmental analysis to evaluate the feasibility of partially replacing Portland cement with oil palm kernel shell ash.

## Figures and Tables

**Table 1:** Calcination temperature for six samples, after drying at 100 °C for 24 h, cutting in an electric crusher, grinding in a jaw mill, and subsequent crushing in a ball mill.

Calcination Temperature (°C)	Drying at 100 °C for 24 h in an Oven. Cutting in an Electric Shredder. Grinding in a Jaw Crusher. Calcination in a Muffle Furnace for 1.5 h. Crushing in a Ball Mill for 1 h
**Drying a 100°**	MO-T100C-C-t24 h-c-m
**500**	M17A-c-m-T500C-t1.5h-tr1h
**600**	M18A-c-m-T600C-t1.5h-tr1h
**700**	M19A-c-m-T700C-t1.5h-tr1h
**800**	M20A-c-m-T800C-t1.5h-tr1h
**900**	M21A-c-m-T900C-t1.5h-tr1h
**1000**	M22A-c-m-T1000C-t1.5h-tr1h

c = Cutting in an electric crusher, T = temperature, C = calcination (°C), t = time, tr = crushing in a ball mill, M = grinding in a manual jaw crusher.

**Table 2:** Results of analyzed samples in percentages.

Chemical Composition (%)	Result (%)						
	MO-T100C-C-t24 h-c-m	M17A-c-m-T500C-t1.5h-tr1h	M18A-c-m-T600C-C-t1.5h-tr1h	M19A-c-m-T700C-C-t1.5h-tr1h	M20A-c-m-T800C-C-t1.5h-tr1h	M21A-c-m-T900C-C-t1.5h-tr1h	M22A-c-m-T1000C-C-t1.5h-tr1h
**Na_2_O**	-	0.13	-	0.04	0.14	-	-
**MgO**	3.59	3.16	3.41	3.06	3.05	3.15	3.19
**Al_2_O_3_**	2.30	9.97	9.39	10.82	7.57	7.78	4.11
**SiO_2_**	**78.38**	**69.51**	**71.24**	**68.47**	**72.17**	**73.17**	**76.80**
**P_2_O_5_**	4.22	3.38	3.65	3.14	3.15	3.30	3.48
**SO_3_**	0.94	0.83	0.69	1.12	0.97	0.81	0.88
**K_2_O**	4.69	5.50	4.53	6.14	5.46	5.24	5.49
**CaO**	3.88	4.78	4.04	3.30	3.54	3.23	3.30
**TiO_2_**	0.24	0.26	0.29	0.22	0.30	0.26	0.29
**MnO**	0.12	0.10	0.11	0.08	0.09	0.10	0.10
**Fe_2_O_3_**	1.65	2.37	2.65	3.60	3.69	2.96	2.36

**Table 3:** Comparison of the chemical composition of POFA across different studies.

Product	Procedure for Obtaining the Ash	Calcination Temperature °C	% SiO_2_	% Al_2_O_3_	% Fe_2_O_3_	References
***POFA*** **(MDEF-c-m-T500C-t1.5h-tr1h-T600C-t2h)**	Drying at 100 °C for 24 h in an oven. Cutting in an electric crusher. Grinding in a jaw mill. Calcination in a muffle furnace at 500 °C for 1.5 h. Crushing in a ball mill for 1 h. Second calcination at 600 °C (2 h).	First calcination at 500 °C and second calcination at 600 °C	66.57	5.50	2.57	Own study (Standardized sample whose process is presented in the following sections of this article)
**POFA-FB**	Incineration (1 h), sieving (45 mm), crushing	60	64.72	2.47	1.39	[55]
**POFA-K**	Incineration (1 h), sieving (45 mm), crushing	150	64.08	2.79	2.09	
**G-POFA**	Dehydration at 105 ± 5 °C (24 h), sieving, ball milling, calcination (1.5 h)	500 ± 50	51.18	4.61	3.42	[56,57]
**U-POFA**	Dehydration at 105 ± 5 °C (24 h), sieving, ball milling, calcination (1.5 h), grinding to 2 µm	500 ± 50	65.02	5.73	4.42	
**G-POFA**	Dehydration at 105 ± 5 °C (24 h), sieving, calcination (2 h)	600	59.17	3.73	6.33	[37,58]
**T-POFA**	Dehydration at 105 ± 5 °C (24 h), sieving, grinding, calcination (2 h), grinding	600	69.03	3.10	4.34	
**POFA**	Drying, sieving, grinding	It does not present calcination data	50	0.7	2	[38]
**POFA**	Grinding and calcination	600	58.1	2.6	1.67	[59,60]
**POFA**	Crushing and ball milling	It does not provide calcination data	57.8	2.3	9.6	[42]
**MT-POFA**	Calcination (2 h), grinding	600	66.24	3.52	3.93	[61]
**POCP**	Washing, drying at 110 °C, sieving, grinding	It does not provide calcination data	59.9	5.37	6.93	[32]
**POFA**	Washing, drying at 100 °C (24 h)	It does not provide calcination data	52.8	2.68	4.45	[62]
**G-POFA**	Drying at 100 °C (24 h), grinding	It does not provide calcination data	53.3	1.9	1.9	[63]
**POFA**	Grinding, drying at 100 °C (24 h), grinding in a rotary drum	800 a 1000	63.4	5.51	4.2	[64]
**POFA**	Calcination, drying at 105 °C (24 h), sieving, grinding	It does not provide calcination data	64.17	3.73	6.33	[16]
**POFA**	Grinding (2 h)	It does not provide calcination data	43.6	8.5	10.1	[65]
**POFA**	Drying at 100 °C (24 h), grinding, calcination (2 h)	600	37.04	18.89	3.59	[66]
**POFA**	Calcination and grinding	750	45.88	0.64	1.90	[67]

**Table 4:** Determination of the percentage of organic material for the samples calcined at temperatures ranging from 500 °C to 1000 °C.

Sample Coding	Calcination Temperature (°C)	Organic Material Losses (%)
MO-T100C-C-t24 h-c-m	Secado a 100 °C	14.5 (humedad)
M17A-c-m-T500C-t1.5h-tr1h	500	72.60
M18A-c-m-T600C-t1.5h-tr1h	600	**70.36**
M19A-c-m-T700C-t1.5h-tr1h	700	72.55
M20A-c-m-T800C-t1.5h-tr1h	800	74.72
M21A-c-m-T900C-t1.5h-tr1h	900	79.03
M22A-c-m-T1000C-t1.5h-tr1h	1000	81.33

**Table 5:** Analysis of elements contained in the ashes for the ash calcined at 600 °C (M18A-c-m-T600C-t1.5h-tr1h).

Elements	Weight%
C	86.29
O	12.19
Si	0.90
K	0.62
**Total**	100.00

**Table 6:** Elemental analysis of the contents in the ashes for the sample with the standardized process (MDEF-c-m-T500C-t1.5h-tr1h-T600C-t2h).

Elements	Weight%
O	49.45
Si	49.49
K	0.65
Ca	0.42
**Total**	100.00

**Table 7:** Analysis of the element content in the POFA with the standardized process and calcination at 650 °C for LOI analysis.

Elements	Weight %
O	45.01
Si	54.45
K	0.54
**Total**	100.00

**Table 8:** Analysis of the element content in the POFA with the standardized process and calcination at 750 °C for LOI analysis.

Element	Weight%
O	34.63
Al	0.57
Si	62.67
K	1.08
**Total**	100.00

**Table 9:** Chemical composition of the standardized sample MDEF-c-m-T500C-t1.5h-tr1h-T600C-t2h through XRF analysis.

Chemical Compound	Composition *w*/*w*
SiO_2_	66.57
TiO_2_	0.38
Al_2_O_3_	5.50
Fe_2_O_3_	2.57
Mn_3_O_4_	0.21
MgO	3.36
CaO	5.73
Na_2_O	0.41
K_2_O	8.73
P_2_O_5_	4.51
SO_3_	0.41
Cr_2_O_3_	0.14
NiO	0.14
CuO	0.09
ZnO	0.11
SrO	0.08
CeO_2_	0.32
SnO_2_	0.26
Cl	0.51

## Data Availability

The original contributions presented in this study are included in the article. Further inquiries can be directed to the corresponding author.
